# The impact of gender stereotypes on physical education lessons: a pilot study regarding the qualitative analysis of teachers’ perceptions

**DOI:** 10.3389/fpsyg.2025.1575686

**Published:** 2025-04-23

**Authors:** Anamaria Pautu, Simona Petracovschi, Martin Domokos

**Affiliations:** Department of Physical Education and Sport, West University of Timisoara, Timișoara, Romania

**Keywords:** gender stereotypes, teacher attitudes, physical education, sentiment analysis, student performance, educational practices

## Abstract

**Introduction:**

This study examines the feelings of physical education teachers toward gender stereotypes and their impact on how boys and girls are perceived and supported during physical education classes. It explores how teachers’ expectations and attitudes reflect cultural biases, such as associating boys with physically demanding sports and girls with activities emphasizing grace and flexibility.

**Methods:**

Semi-structured interviews were conducted with eight teachers from diverse age groups and work environments (urban and rural). The research utilized MAXQDA software to analyze the emotional tone of the responses, categorizing them as positive, neutral, or negative.

**Results:**

The findings reveal that teachers generally maintain a neutral tone when discussing gender differences, focusing on objective observations rather than emotional evaluations. However, instances of positive attitudes, such as appreciating girls’ discipline and performance, suggest efforts to challenge stereotypes. In contrast, some responses reflect stricter expectations or criticisms of girls, which can perpetuate negative stereotypes.

**Conclusion:**

Teachers’ feelings significantly influence how they address gender stereotypes in physical education. Positive sentiments are associated with encouragement and inclusivity, while neutral or negative attitudes often reinforce traditional expectations, allowing stereotypes to persist. These findings underscore the need for targeted training programs to help teachers develop equitable and inclusive practices that reduce the influence of gender stereo-types on students’ experiences in physical education.

## Introduction

Gender stereotypes represent a significant challenge in education, profoundly impacting students’ development and performance. In the context of physical education, these stereotypes often manifest through different expectations for boys and girls, influencing how they are perceived, assessed, and encouraged to participate in sports activities (Messner, 2011; Brown and Evans, 2004; Connell, 2002). Over time, the role of physical education teachers has expanded, becoming not only facilitators of sports activities but also agents of social change, responsible for creating an inclusive and equitable learning environment. Reducing gender stereotypes has thus become an essential component of pedagogical practices, contributing to the development of an educational culture that pro-motes equal opportunities and mutual respect (Hargreaves, 2014; Guerrero and Puerta, 2023; Hall, 2004; Gard, 2008).

By its very nature, physical education is a field where physical differences and gender stereotypes can be more visible and pronounced. Traditionally, boys have been perceived as more suitable for team sports and activities requiring strength and competition, while girls have often been associated with sports emphasizing grace and flexibility, such as gymnastics or dance (Vertinsky, 1992; Olafson, 2002; Clark and Paechter, 2007). These stereotypes, deeply rooted in popular culture and traditional educational systems, can limit students’ potential, reduce their self-confidence, and restrict their access to diverse opportunities for personal and professional development (Azzarito and Solomon, 2005; Kirk, 2010).

Physical education teachers, as educational leaders, play a major role in counteracting these stereotypes and creating a learning environment that encourages all students, regardless of gender, to develop their skills and actively participate in all aspects of physical education (Hills, 2007; Lenskyi, 1994; Penney, 2002). Research shows that educational interventions addressing gender stereotypes can positively impact students’ engagement in sports activities and their overall development (Flintoff and Scraton, 2001; Wright, 2001; Azzarito and Solomon, 2005). By adopting gender-sensitive pedagogical strategies, teachers can contribute to creating a learning environment where all students feel valued and supported. These strategies include, but are not limited to, providing equitable feedback, organizing activities that involve boys and girls equally, and facilitating open discussions about gender and equality (Messner, 2011; Waddington et al., 1998; Clark and Paechter, 2007).

It is also very important for teachers to be aware of their own attitudes and behaviors, which can influence students’ perceptions and perpetuate gender stereotypes. Furthermore, research highlights the importance of continuous teacher training in gender equality and diversity, providing them with the necessary tools to effectively manage gender dynamics in their classrooms (Hargreaves, 2007; Guerrero and Puerta, 2023). Initial training and ongoing professional development should include specific modules addressing the impact of gender stereotypes in physical education, offering practical examples and intervention strategies (Tinning, 2010). This way, teachers can be better equipped to recognize and combat gender stereotypes, contributing to the development of a more equitable and inclusive educational environment (Vertinsky, 1992).

However, reducing gender stereotypes in physical education is not an easy task. It requires a constant commitment from teachers to reflect on their own practices and adapt teaching methods according to the needs and characteristics of each student (Lenskyi, 1994; Ennis and Lazarus, 1990). Additionally, this effort involves close collaboration with other educational stakeholders, including parents, school administration, and the local community, to ensure a consistent approach (Hills, 2007; Hastie and Siedentop, 2006). Through joint efforts and a commitment to promoting gender equality, physical education teachers can significantly contribute to developing a school culture that values diversity and encourages all students to reach their full potential (Liu, 2012; Dagkas and Armour, 2012).

Therefore, reducing gender stereotypes in physical education is not only a matter of social justice but also of educational effectiveness. By recognizing and addressing these stereotypes, teachers can create a more equitable and inclusive learning environment that supports the holistic development of every student. In this context, it is essential to constantly review and adapt pedagogical practices to reflect the values of equality and respect that underpin quality education (Flintoff and Scraton, 2001; Pang and Lee, 2008; Duda, 1991).

In Romania, research on the influence of gender stereotypes in physical education is still in its infancy, and teachers’ perspectives have not been fully investigated. Existing studies suggest that stereotypes influence how students participate in physical education classes, and teachers play a very important role in maintaining or reducing these stereotypes. For example, comments suggesting distinct activities for boys and girls, such as encouraging girls to avoid football or to be more reserved, can limit their participation in activities considered masculine and create obstacles to their development. Additionally, teachers’ perceptions, such as viewing girls as more organized and attentive and boys as more skilled in technical sports and sciences, contribute to the segregation of educational choices based on gender (Proca and Gall, 2018).

Gender stereotypes tend to generate differences in how students are treated during sports activities, perpetuating the idea that more demanding or competitive sports are “masculine,” while activities perceived as easier, or esthetic are “feminine.” This dynamic limit equal participation opportunities for both genders, reinforcing inequalities in access to physical and sports activities. Understanding teachers’ opinions on gender stereotypes and their perspectives is essential for addressing educational inequalities in Romania. Although international studies highlight the importance of physical education teachers in shaping and maintaining gender stereotypes, research on this topic in Romania is still limited and lacks detail (Proca and Gall, 2018). Most studies focus on general aspects of gender equality in education and do not sufficiently address how physical education teachers think, feel, and act regarding these stereotypes. Specifically, little is known about how teachers perceive their role in combating stereotypes, the methods they use, or the obstacles they face. Moreover, there is insufficient data on differences between urban and rural environments, or how teachers’ age or experience might influence their views and behaviors concerning these stereotypes.

Therefore, there is a gap in the Romanian literature regarding how physical education teachers influence (positively or negatively) gender stereotypes in their classes. This study aims to fill this gap by directly analyzing the opinions, attitudes, and teaching methods of physical education teachers. Special attention will be given to differences related to the teachers’ backgrounds (urban/rural), gender, and age to better understand how gender stereotypes are created and can be overcome in physical education classes. Thus, the research will contribute to advancing knowledge in the field and offer practical suggestions for teacher training and continuous development to achieve a more equitable and inclusive physical education system in Romania. Given the exploratory nature of this research, this study serves as a pilot study to assess initial trends in teachers’ perceptions regarding gender stereotypes in physical education.

## Materials and methods

The data were collected through the structured interview method, an approach suitable for research aimed at obtaining structured and comparable responses. According to Patton (2002), this technique facilitates the comparison of responses from different participants while also capturing each individual’s unique perspective. The choice of the structured interview was based on its advantage of steering discussions toward clearly defined topics, reducing ambiguity, and facilitating response comparisons (Bryman, 2016).

To investigate the perceptions of physical education and sports teachers regarding gender stereotypes, a qualitative methodology based on semi-structured interviews was employed. This approach was chosen as it allows for an in-depth exploration of teachers’ opinions and experiences, providing a detailed perspective on how gender stereotypes influence both teaching practices and student interactions. Given the exploratory nature of this research, this study serves as a pilot study aimed at identifying initial trends in teachers’ perceptions regarding gender stereotypes in physical education. As such, it does not aim to generate generalizable conclusions, but rather to explore emerging patterns and inform future research design. The findings will contribute to shaping future larger-scale research on this topic.

The structure of the interview questions was adapted from Constantinou (2008), who developed a gender stereotype questionnaire for physical education settings, examining aspects such as corrective feedback, teachers’ expectations of boys and girls, and students’ engagement levels.

The first section, “Professional Background,” included questions about age, work environment, and professional experience, aiming to identify potential correlations between these factors and teachers’ perceptions of gender. The second section, “Gender Stereotypes,” aimed to identify possible differences in how teachers provide feedback to students, their expectations of girls and boys, and their application of disciplinary rules. This section also included questions about teachers’ perceptions of physical activity and students’ level of involvement based on gender. The third section, “Student Attitudes,” investigated their behaviors during physical education classes, such as the tendency to segregate by gender, girls’ or boys’ reluctance to participate in certain activities, and the influence of teachers’ perceptions on students’ motivation. The final section, “Inclusion and Diversity,” assessed teachers’ perspectives on diversity in the educational environment, identifying barriers to promoting inclusion and the extent to which schools implement initiatives to reduce gender inequalities.

In this pilot study, eight physical education and sports teachers (four women and four men) were interviewed, aged between 27 and 66, coming from localities of varying sizes, both urban and rural. The selection of participants was made to include one woman and one man in each of the four targeted age categories: 25–35, 35–45, 45–55, and over 55 years. The interviews were conducted exclusively with teachers working in school-based physical education, without including other specialists or educators from related fields. The interview analysis was carried out using MAXQDA software, which facilitated the coding of responses and the identification of sentiment patterns expressed by participants—positive, negative, or neutral. This analysis enabled the identification of preliminary trends in perceptions regarding gender stereotypes and how these may influence teaching practices, providing insights relevant for the development of future, larger-scale investigations. These findings align with previous research, such as that of Constantinou (2008), which highlights the existence of differentiated feedback and distinct expectations based on gender in physical education classes.

Among them, A.T. comes from a small urban town with a population of 10,627 (National Institute of Statistics, 2021). L.S. teaches in a small rural locality with 2,833 inhabitants (Moșnița Nouă Commune Hall, 2025a). M.M. teaches in a rural locality with a population of approximately 5,000–6,000 (Biled Commune Hall, 2025). From the urban environment, B.D., C.A., H.C., and G.F. teach in a large city with around 350,000 inhabitants (National Institute of Statistics, 2021), while C.C. teaches in a village, part of a commune, with approximately 1,590 inhabitants (Moșnița Nouă Commune Hall, 2025b).

Among the participants, four come from small towns with populations ranging from 1,590 to 10,627, while five come from larger urban areas with populations between 350,000 and 5,000-6,000. This distribution reflects a diversity of urban and rural environments, which, in the context of a pilot study, was essential to capturing a broad range of initial perspectives on gender stereotypes. The interview guide, developed in line with the research objectives, included questions designed to capture teachers’ perceptions of gender stereotypes, their impact on professional activity, and the emotions experienced in relevant situations.

Due to the diversity of the selected teachers, we did not receive similar responses to the questions addressed. Each teacher applied different teaching styles and approaches to gender-related topics in the classroom, reflecting their unique experiences, pedagogical strategies, and perspectives on gender issues in physical education (Constantinou, 2008).

For analyzing the collected data, MAXQDA software was used, recognized for its efficiency in qualitative and mixed-methods data analysis. This software enables the visualization of patterns in the data and the systematic analysis of emotional expressions, making it suitable for sentiment analysis and thematic exploration (Kuckartz and Rädiker, 2019). Sentiment analysis was employed to evaluate the emotional tone of the responses, categorizing them as positive, negative, or neutral. This method highlights both the explicit opinions of participants and the intensity of their emotional perceptions (Pang and Lee, 2008).

The use of MAXQDA facilitated the identification of responses expressing dissatisfaction or frustration with gender stereotypes, as well as those indicating acceptance or appreciation. The software enabled mapping the evolution of emotions based on themes and contexts, contributing to a deeper understanding of the subjective experiences of teachers (Liu, 2012). Thus, sentiment analysis allowed researchers to capture not only opinions but also the intensity of expressed emotions, aligning with recommendations in the specialized literature regarding the importance of the subjective dimension in studies of this type (Cambria et al., 2017; Bryman, 2016).

Sentiment analysis, also known as “opinion mining,” is a qualitative method used to evaluate the emotional tone of texts and to understand the attitudes, feelings, and opinions expressed. Initially developed by Bing Liu (2012), this approach allows for the classification of sentiments into categories such as positive, negative, and neutral, highlighting how certain topics or events are perceived. The method has rapidly evolved across various fields, including social sciences and education, due to its ability to reveal not only general opinions but also the intensity of sentiments. In educational contexts, sentiment analysis can shed light on subtle attitudes and perceptions related to themes such as gender equality, providing insights into how teachers and students emotionally respond to these issues (Pang and Lee, 2008).

This method is also valuable for identifying the emotional factors influencing participants’ opinions and behaviors, even when data are collected at a single point in time. By evaluating expressed emotions, researchers can highlight both the frequency of positive or negative opinions and the degree of their intensity (Maynard and Funk, 2011). In research focused on physical education, tools such as MAXQDA facilitate the coding and classification of sentiments, enabling the integration of thematic analyses with sentiment evaluation (Kuckartz and Rädiker, 2019). Thus, qualitative analysis software can emphasize emotions and associate them with the researched themes, helping us better understand how participants’ perceptions and feelings are shaped by their experiences and beliefs. These findings, though limited in scope, support the relevance of sentiment analysis in exploratory, pilot-stage research on gender in education (Table 1).

**Table 1:** Categories and descriptions of sentiments identified in teachers’ responses regarding gender stereotypes.

Category	Description
Neutral	Text segments marked as “neutral” indicate that teachers discussed gender stereotypes or gender differences in a descriptive manner, without expressing a positive or negative opinion. These segments focus on factual observations without adding emotional or evaluative connotations.
Positive	Segments marked as “positive” reflect a favorable perception or a positive attitude toward managing or reducing gender stereotypes. These segments highlight teachers’ positive efforts to combat stereotypes or to promote gender equality.
Negative	Segments marked as “negative” indicate a less favorable perception of the behavior or performance of girls or boys. These segments may reflect a more critical attitude or greater strictness toward a particular gender, suggesting a negative perception or frustration related to certain stereotypes or behaviors.

## Results

The analysis of interviewees’ responses suggests a predominantly favorable perception and a positive sentiment toward girls in terms of behavior and academic performance compared to boys. This tendency, observed in this pilot study, becomes evident in the positive and neutral tones of teachers’ responses, especially when referring to girls’ discipline and compliance with academic expectations. Positive sentiments are emphasized by teachers’ appreciation of the fact that girls are more likely to follow rules and meet school requirements, a perception that may influence how they provide feedback and interact with them. For instance, G.F. (66 years old, urban environment) expresses a positive sentiment when stating, “I have always felt that at the junior level (13/14 years old), girls outperform boys,” suggesting that girls exceed boys in terms of performance. G.F. also adds that “in the beginning, girls are more active,” reinforcing the positive perception of their engagement in sports activities (Table 2).

**Table 2:** Summary of teachers’ perceptions and expectations of girls and boys, based on gender, age, and work environment.

Document	Responses	Codes	Work environment	Gender	Age	Sentiment
G.F.—Female (66 years)—Urban	G.F. expresses a clearly positive perception of girls, stating that they outperform boys in the early stages of physical and academic development: “I’ve always felt that at the junior level (13/14 years old), girls are ahead of boys.” She also notes that girls in her teams are ambitious and actively participate in all sports, reinforcing the positive sentiment: “No, the girls in my teams are very ambitious.”	… > Positive	Urban	Female	66	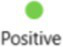
C.A.—Female (45 years)—Urban	C.A. has different expectations for boys and girls but expresses an empathetic tone toward girls, suggesting a positive sentiment: “It’s harder with girls. I have higher expectations for boys in sports.” She mentions that girls are more stubborn but sees it as a challenge rather than a major issue: “I’m stricter with girls because they are more stubborn and think they know everything.”	… > Positive/Neutral	Urban	Female	45	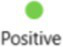
B.D.—Female (43 years)—Urban	B.D. has a positive perception of girls’ participation in physical education, especially up to the 7th grade: “There are no gender stereotypes, and I believe all children should do all sports.” However, she acknowledges that girls become more hesitant after the 7th grade in certain activities, adding a neutral element: “After the 7th grade, yes, when it comes to gymnastics or things like somersaults and splits, they are a bit hesitant because boys are watching.”	… > Positive	Urban	Female	42	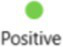
C.C.—Male (47 years)—Rural	C.C. expresses a balanced perception, mentioning that he has the same academic expectations for boys and girls but higher physical expectations for boys: “In terms of results, yes, I have the same expectations. Physically, I have higher expectations of boys than girls.” He also notes that boys are more reluctant about gymnastics, reinforcing a neutral sentiment.	… > Neutral	Rural	Male	47	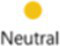
H.C.—Male (63 years)—Urban	H.C. expresses a neutral sentiment with positive notes, mentioning that boys’ and girls’ performances differ but that some girls can outperform boys: “Yes, there are girls who are better than boys.” Additionally, he observes that boys are more undisciplined but does not see it as a major issue, indicating a balanced perception: “The truth is they mess around more, so they are more undisciplined in my experience.”	… > Neutral	Urban	Male	63	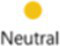
M.M.—Male (40 years)—Rural	M.M. acknowledges that certain sports activities suit boys better but does not make significant differences in disciplinary rules: “Regarding discipline, yes, but we have some rules where girls have priority.” The general sentiment is neutral, with some positive notes: “There are moments when girls are better than boys if we are playing handball.”	… > Neutral	Rural	Male	40	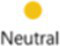
L.S.—Female (33 years)—Rural	L.S. expresses a clear desire to reduce gender separation among students and works to correct this behavior, showing a positive attitude toward gender equality: “Yes, I do not understand why they do this. I’m working on it and do not agree.” Additionally, L.S. observes no major differences in boys’ and girls’ physical activity, reinforcing an equitable perception: “Girls are just as active.”	… > Positive	Rural	Female	33	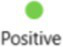
A.T.—Male (27 years)—Urban	A.T. emphasizes that there should be no differences between boys and girls in physical education classes, stating: “I do not think there should be any, especially among children, and they should understand their equality in PE class.” However, he acknowledges that for more physically demanding tasks, he chooses boys, reflecting a neutral sentiment: “I choose the boys because they are better developed.”	… > Neutral	Urban	Male	27	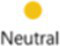

In contrast, when boys are discussed, some responses reveal a more reserved tone or even slight frustration typically expressed through neutral or cautiously critical sentiments. C.C. (47 years old, rural environment) conveys a neutral sentiment when stating, “Physically, I have higher expectations of boys than girls.” This statement seems to reflect a neutral attitude, lacking the same enthusiasm present when discussing girls’ performance. Additionally, C.C. observes that “as students get older, boys develop physically faster than girls,” but acknowledges that in the early school years, girls face no difficulty competing at the same level as boys, thus maintaining a balanced and neutral tone.

### Gender as an influencing factor

The gender of teachers may influence how they formulate their expectations and sentiments toward male and female students. According to the responses collected in this pilot study, female teachers generally exhibit a positive attitude and sentiment regarding the differences between boys and girls. For example, C.A. (45 years old, urban environment) expresses her feelings empathetically, mentioning that “it’s harder with girls. I have higher expectations of boys in sports,” which may suggest that although boys are perceived as more suitable for sports activities, girls are viewed with greater empathy and perhaps even protection. This may indicate a stronger emotional involvement in the performance of girls, with C.A. adding, “I’m stricter with girls because they are more stubborn and think they know everything,” implying a perceived need for improving girls’ performance.

On the other hand, male teachers in this sample tend to express a more neutral or even critical perception of boys. For instance, M.M. (40 years old, rural environment) observes that “some things suit boys better,” but also remarks that “in general, girls are a bit more attentive,” expressing a neutral yet slightly critical sentiment toward boys.

Female teachers who participated in this study often express their expectations and feelings toward students in a direct manner, emphasizing the positive qualities of both genders. L.S. (33 years old, rural environment), with a positive and egalitarian attitude, states: “I have expectations from all my students. Yes, I have the same expectations,” suggesting a sense of equality between boys and girls regarding the demands imposed on them.

### Age and experience

Teachers’ ages appear to influence how they perceive and express sentiments about differences between boys and girls. Older teachers with extensive experience in this study tend to express neutral or positive sentiments, indicating a possible acceptance of gender differences as natural and inevitable. For example, H.C. (63 years old, urban environment) conveys a neutral yet open sentiment, stating that “performance and norms differ between boys and girls. Yes, there are girls who are better than boys.” This sentiment of acceptance reflects a non-judgmental approach, supported by vast experience in education.

Younger teachers, such as A.T. (27 years old, urban environment), adopt a more flexible and adaptable approach, maintaining a neutral but slightly positive attitude. A.T. suggests that “I clearly have higher physical expectations from boys […] for girls, I expect them to be perseverant,” indicating an openness to each student’s individuality without rigid distinctions based on gender.

### Work environment (urban vs. rural)

The environment in which teachers work also appears to influence how they formulate their expectations and sentiments toward students. Teachers from urban areas in this pilot sample express a more positive sentiment regarding gender differences, often emphasizing that girls adapt better to educational requirements. For example, B.D. (43 years old, urban environment) conveys a positive sentiment, stating, “In gymnastics, I do not have the same expectations for boys as for girls,” suggesting that girls excel in certain areas, while boys are evaluated more leniently.

In contrast, teachers from rural areas, such as M.M. (40 years old, rural environment), maintain a neutral or even slightly negative sentiment regarding gender differences, mentioning that “some things I can say suit boys better.” This perspective might reflect a more pragmatic or traditional approach, where the focus is placed on practical approaches rather than gender differences.

The analysis of responses indicates that gender, age, and work environment influence not only teachers’ perceptions but also their sentiments toward differences between boys and girls. However, given the exploratory nature of this pilot study, these observations should be interpreted as initial trends rather than general conclusions. Female teachers, those from urban environments, and those with longer teaching experience tend to express a more positive sentiment toward girls’ performances. In contrast, younger teachers and those from rural areas displayed more neutral perspectives, but also greater flexibility in evaluating students (Table 3).

**Table 3:** Analysis of Teachers’ Sentiments Regarding Gender Stereotypes.

Document	Responses	Sentiment	+ Words	− Words	Difference
G.F.—Female (66 years)—Urban	“I have always felt that at the junior level 3, girls outperform boys. Boys start catching up from junior level 2.”	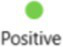	2	0	2
C.A.—Female (45 years)—Urban	“I’m stricter with girls because they are more stubborn and think they know everything.”	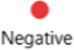	0	1	−1
B.D.—Female (43 years)—Urban	“In gymnastics, I do not have the same expectations for boys as for girls. For girls, I have higher expectations. For team sports, I have the same expectations, but for gymnastics, unfortunately, it’s not the same.”	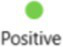	9	1	8
C.C.—Male (47 years)—Rural	“Most of them do not have this stereotype, but there’s a small percentage, maybe 5 to 10%, who are hesitant about certain games they perceive differently.”	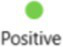	4	3	1
H.C.—Male (63 years)—Urban	“Yes, naturally, as performance and norms differ between boys and girls. Yes, there are girls who are better than boys.”	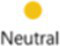	1	1	0
M.M.—Male (40 years)—Rural	“There are moments when girls are better than boys in handball. But in football, they do not quite match up. As boys grow taller and stronger, they surpass them. Up to a certain point or age, girls can match or even surpass boys. But I avoid playing boys against girls.”	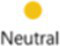	3	3	6
L.S.—Female (33 years)—Rural	“I’ve always tried to adapt exercises to include everyone. (…) In sports games, every boy and girl has to go through the same exercises to learn as much as possible about sports. No, I do not tell them this is for boys or girls. It’s a sports game, and we need to learn all of them.”	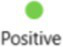	3	0	3
A.T.—Male (27 years)—Urban	“I may have higher physical expectations for boys, meaning they should handle a higher level of effort than girls.”	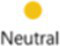	3	3	0

The interpretation of this table, which includes evaluations of sentiment (neutral, positive, and negative) and additional observations for each interview segment, provides an overview of teachers’ attitudes toward gender stereotypes. By associating content codes with the emotional tone of the responses, researchers can better understand the level of approval, disapproval, or neutrality in teachers’ discourse.

This interpretation provides a preliminary perspective on how teachers appear to perceive and discuss gender stereotypes in the context of physical education. The table emphasizes a range of perceptions and approaches by teachers regarding gender stereotypes, with text segments marked as “neutral,” “positive,” or “negative,” depending on their tone. These markings help us gain an initial understanding of how each teacher manages their perceptions of gender and how these are reflected in their educational practices. In summary, there is a general tendency to discuss gender stereotypes in an objective and descriptive manner, but with moments where teachers adopt a positive and progressive attitude, particularly when speaking about their efforts to promote gender equality (Table 4).

**Table 4:** General interpretation of sentiment categories (short version).

Category	Description
Neutral	Text segments marked as “neutral” indicate that teachers discussed gender stereotypes or gender differences in a descriptive manner, without expressing a positive or negative opinion.
Positive	Segments marked as “positive” reflect a favorable perception or a positive attitude regarding the management or reduction of gender stereotypes.
Negative	Segments marked as “negative” indicate a less favorable perception of the behavior or performance of girls or boys.

### Positive sentiment—open and equality-oriented attitudes

The positive sentiments expressed by teachers regarding gender stereotypes suggest an open and progressive approach to gender equality in physical education. For example, B.D. (female, 43 years old, urban environment) mentions that there is a difference in expectations for boys and girls but emphasizes that this is an issue that needs to be addressed. The term “unfortunately,” which she uses in the context of gymnastics, suggests reflection on these differences and a sense of regret, coupled with a desire for change. This can be interpreted as a positive attitude that demonstrates an intention to overcome gender stereotypes and promote equality in sports activities. Similarly, C.C. (male, 47 years old, rural environment) highlights that most of his students are not influenced by gender stereotypes, which suggests a positive perception of progress made in combating them. Although he mentions slight resistance among a small percentage of students (between 5% and 10%), this does not diminish his overall positive perception. Likewise, L.S. (female, 33 years old, rural environment) stands out with her active approach to combating gender stereotypes, ensuring that every student, regardless of gender, goes through the same exercises. She makes no distinction between boys and girls, denoting an egalitarian and proactive attitude essential for overcoming stereotypes and promoting inclusive physical education. Similarly, G.F. (female, 66 years old, urban environment) expresses appreciation for the performance of girls compared to boys, emphasizing that junior girls are “ahead of boys” up to a certain level. This demonstrates support for and recognition of girls’ abilities in sports, fostering a positive perception of them.

### Negative sentiment—criticism of girls and perpetuation of prejudices

In contrast to positive approaches, negative sentiment toward gender stereotypes appears in the comments of C.A. (female, 45 years old, urban environment), who expresses a stricter attitude toward girls’ behavior. She considers that girls are “more stubborn” and “think they know everything,” suggesting a negative perception of their behavior. This may reflect internalized stereotypes and stricter expectations applied to girls, which could reinforce negative stereotypes that impact their development in physical activities. Such strictness toward girls might contribute to the strengthening of preconceived notions about their behavior and lead to less equitable education. Thus, the negative sentiment expressed by C.A. may indicate frustration with the expectations and behaviors she perceives during physical education classes, potentially negatively affecting her relationship with students and the overall classroom atmosphere.

### Neutral sentiment—objective observations and lack of emotional involvement

Neutral sentiments express an objective and balanced observation of gender differences in physical activities, without positive or negative connotations. H.C. (male, 63 years old, urban environment) observes that there are performance differences between boys and girls but emphasizes that some girls are better than boys. Similarly, M.M. (male, 40 years old, rural environment) notes that girls can outperform boys in certain games, but in other activities, such as football, boys tend to be more physically adept. Both observations are made in a neutral tone, without suggesting that these differences should be changed or that they represent a problem. These comments reflect a pragmatic view of the realities in physical education settings, where performance differences between genders are acknowledged but not emotionally evaluated. These segments, therefore, highlight perceptions based on facts and direct observations, unaccompanied by value judgments or intentions for change.

Recent research highlights the persistence of gender stereotypes in physical education, even as policies and curricula strive to promote gender equity. A systematic review found that educational materials, such as physical education textbooks, continue to reinforce traditional gender norms, portraying boys as active and competitive while associating girls with esthetics and passivity (Karisman et al., 2025). Moreover, a large-scale study conducted in Spain on physical education teachers and students revealed a discrepancy between teachers’ intentions and students’ perceptions of gender equity. While many teachers believe they are fostering an inclusive environment, students often experience subtle biases that reinforce traditional expectations (Castro-García et al., 2025). Furthermore, gender-sensitive pedagogical approaches are necessary to counteract these biases, especially through teacher training programs that incorporate critical feminist perspectives (Preece and Bullingham, 2020). Studies show that intervening at the pre-service teacher level is crucial to dismantling implicit gender biases before they become ingrained in teaching practices (Avraam and Anagnostou, 2022). By integrating these findings into teacher training and curriculum development, physical education can become a truly equitable space where students are encouraged to explore diverse sports without gender-based restrictions.

## Discussion

### Openness to gender equality (positive sentiment)

Teachers with a positive attitude toward gender equality in physical education may strive to create an inclusive environment where boys and girls are treated equally. Flintoff and Scraton (2001) demonstrated that teachers adopting such an approach can help reduce gender stereotypes. Moreover, Guerrero and Puerta (2023) observed that, despite the assumption that rural teachers are more traditional, they can adopt more balanced practices due to training programs and experience exchanges. This tendency appears in the current pilot study as well, where some teachers from rural areas seemed to express openness to promoting equality. The studies by Kollmayer et al. (2018) and Hargreaves (2014) suggest that continuous training and support from schools can help teachers improve their practices and actively support gender equality.

### Resistance to change and perpetuation of stereotypes (negative sentiment)

Teachers with a negative attitude toward gender equality believe that boys are more suited for physical activities than girls and apply different rules to the two groups. Messner (2011) found that these stereotypes remain prevalent in many schools, even in urban areas, where teachers are presumed to be more open to change. Similarly, Hills (2007) emphasized that, although awareness of the issue has increased, there is still significant resistance to implementing methods that completely eliminate gender differences.

In this pilot study, such attitudes were also noted among some teachers, who appeared to maintain different expectations for boys and girls, potentially limiting girls’ participation in physical activities.

### Apparent neutrality and maintaining the status quo (neutral sentiment)

Teachers adopting a neutral attitude do nothing to change gender stereotypes but also do not actively support them. Hargreaves (2007) observed that such teachers are often aware of the issue of gender equality but take no action to change the situation. In this study, these teachers apply the same rules for all students but sometimes differentiate physical tasks, thereby perpetuating traditional norms without being fully aware of it.

The studies by Tsouroufli (2002) and Anderson and White (2004) show that teachers with neutral attitudes inadvertently contribute to maintaining stereotypes, especially if they lack sufficient support or training. Thus, even if they do not actively oppose gender equality, they also do nothing to encourage progress. Some of the teachers in this pilot study appeared to act similarly: they apply the same rules for all students but occasionally differentiate physical tasks, thereby potentially reinforcing traditional norms without being fully aware of it. A similar pattern emerged in the present pilot study, where neutrality was observed in some teachers’ approach to promoting gender equality. These teachers neither encourage nor combat stereotypes, which may lead to their continued perpetuation among children.

## Conclusion

The sentiment analysis carried out in this pilot study offers a preliminary perspective on how teachers’ age, environment, and gender may influence their perceptions and feelings toward gender stereotypes in physical education. The results, structured according to the research objectives and supported by examples extracted from interviews with teachers, highlight the diversity of opinions and approaches regarding gender stereotypes.

The study results show that gender, age, and environment can influence physical education teachers’ perceptions and attitudes toward gender stereotypes, as well as the differences in expectations for boys and girls in sports activities.

Regarding the first objective, the analysis indicates that teachers’ perceptions of gender stereotypes seem to be influenced by their gender, age, and professional environment. Female teachers generally show greater openness to promoting equality among students, adopting methods aimed at reducing gender-based differences. For example, teacher L.S., from a rural area, mentions that she avoids separating students by gender, promoting an egalitarian approach.

On the other hand, some male teachers acknowledge having different expectations for boys and girls, especially when it comes to tasks that require greater physical effort. This tendency is reflected in the case of A.T., who selects boys for tasks that require higher physical capacity, considering them “better physically developed.”

Teachers’ age also appears to be a relevant factor. Younger teachers, such as A.T. (27 years old), although influenced by traditional stereotypes, show greater openness toward gender equality. In contrast, older teachers, like H.C. (63 years old), tend to adopt a neutral stance, passively accepting gender differences without actively intervening to combat stereotypes.

The professional environment also seems to be relevant. Teachers in urban areas are generally more open to challenging gender stereotypes, likely due to exposure to more diverse educational contexts. For example, teacher G.F., from an urban school, supports gender equality and emphasizes the importance of offering “equal opportunities for all, regardless of gender.” In comparison, teachers from rural areas, such as M.M. (40 years old), adopt a more reserved attitude, perceiving gender differences as a natural reality within physical education classes.

This pilot study suggests that gender, age, and professional environment can influence how teachers perceive and address gender stereotypes in physical education. However, a study on a larger sample is needed to gain a deeper understanding of how teachers’ personal perceptions influence their teaching practices and how these are reflected in their lessons.

Female teachers, younger teachers, and those working in urban environments seem more inclined to promote gender equality and adopt inclusive teaching methods. These factors should be considered in teacher training programs to support the development of equitable and inclusive educational practices that reduce the influence of gender stereotypes on students.

### Teachers’ sentiments toward gender stereotypes

The analysis reveals varied emotional reactions. Some teachers display a neutral sentiment toward stereotypical behaviors, considering them part of “normality” and not requiring intervention. For example, M.M. (40 years old, rural) observes that students quickly form ideas about which sports are “suitable” for each gender, but chooses not to intervene.

Other teachers, such as H.C. (63 years old, urban), actively promote equality and recognize students’ individual performance, stating that “there are girls who are better than boys at some activities.” This attitude reflects a positive sentiment and a desire to reduce the impact of stereotypes on students.

The results suggest that teachers’ sentiments could be a factor influencing how they address gender stereotypes and promote inclusion in physical education.

Teachers who express positive sentiments and demonstrate emotional openness to gender equality are more likely to adopt inclusive educational practices. In contrast, those who adopt a neutral or reserved attitude tend to maintain the existing situation, thereby allowing gender stereotypes to persist in the educational environment.

Given the exploratory nature of the research, this study should be regarded as a pilot study, aiming to identify initial trends in teachers’ perceptions and sentiments toward gender stereotypes in physical education. These findings may serve as a foundation for future large-scale research.

### Practical implications and future directions

The results of this study can guide the development of teacher training programs focused on gender equality and methods for combating stereotypes. These courses should be tailored to the needs of teachers from different environments and age groups.

Additionally, educational policies could encourage collaboration between teachers, parents, and the community to support a shared vision of gender equality. The preliminary findings of this study can contribute to the development of strategies and practical guidelines for physical education classes, ensuring equitable support for every student.

### Exploring new research paths

This pilot study opens the door to new research directions. Future studies could explore in more detail the influence of other factors, such as professional experience or university-level training. Longitudinal studies could also be helpful in observing how teachers’ opinions evolve after participating in training programs.

Another approach would be to compare results obtained in different regions or even in other countries, in order to understand the socio-cultural influences on gender stereotypes. Finally, it would be important to give voice to students, to better understand how they perceive teachers’ attitudes and how these attitudes affect their day-to-day school experiences.

## Data Availability

The raw data supporting the conclusions of this article will be made available by the authors, without undue reservation.
